# Introducing a Custom-Designed Volume-Pressure Machine for Novel Measurements of Whole Lung Organ Viscoelasticity and Direct Comparisons Between Positive- and Negative-Pressure Ventilation

**DOI:** 10.3389/fbioe.2020.578762

**Published:** 2020-10-21

**Authors:** Samaneh Sattari, Crystal A. Mariano, Swathi Vittalbabu, Jalene V. Velazquez, Jessica Postma, Caleb Horst, Eric Teh, Tara M. Nordgren, Mona Eskandari

**Affiliations:** ^1^Department of Mechanical Engineering, University of California, Riverside, Riverside, CA, United States; ^2^BREATHE Center at the School of Medicine, University of California, Riverside, Riverside, CA, United States; ^3^Division of Biomedical Sciences, School of Medicine, University of California, Riverside, Riverside, CA, United States; ^4^CellScale Biomaterials Testing, Waterloo, ON, Canada; ^5^Department of Bioengineering, University of California, Riverside, Riverside, CA, United States

**Keywords:** lung and respiratory mechanics, pressure-volume curve, viscoelasticity, positive and negative pressure ventilation, COVID-19, pulmonary biomechanics

## Abstract

Asthma, emphysema, COVID-19 and other lung-impacting diseases cause the remodeling of tissue structural properties and can lead to changes in conducting pulmonary volume, viscoelasticity, and air flow distribution. Whole organ experimental inflation tests are commonly used to understand the impact of these modifications on lung mechanics. Here we introduce a novel, automated, custom-designed device for measuring the volume and pressure response of lungs, surpassing the capabilities of traditional machines and built to range size-scales to accommodate both murine and porcine tests. The software-controlled system is capable of constructing standardized continuous volume-pressure curves, while accounting for air compressibility, yielding consistent and reproducible measures while eliminating the need for pulmonary degassing. This device uses volume-control to enable viscoelastic whole lung macromechanical insights from rate dependencies and pressure-time curves. Moreover, the conceptual design of this device facilitates studies relating the phenomenon of diaphragm breathing and artificial ventilation induced by pushing air inside the lungs. System capabilities are demonstrated and validated via a comparative study between *ex vivo* murine lungs and elastic balloons, using various testing protocols. Volume-pressure curve comparisons with previous pressure-controlled systems yield good agreement, confirming accuracy. This work expands the capabilities of current lung experiments, improving scientific investigations of healthy and diseased pulmonary biomechanics. Ultimately, the methodologies demonstrated in the manufacturing of this system enable future studies centered on investigating viscoelasticity as a potential biomarker and improvements to patient ventilators based on direct assessment and comparisons of positive- and negative-pressure mechanics.

## Introduction

Pulmonary disease is the leading cause of morbidity and mortality worldwide, with new threats emerging from vaping, rising air pollution, exposure from industrial farming, and worldwide pandemics, such as the infamous lung damaging coronavirus disease 2019 (COVID-19) (Rattue, 2012; Guarascio et al., 2013; Nordgren and Bailey, 2016; Nordgren and Charavaryamath, 2018; Eskandari et al., 2019). COVID-19 has risen to be one of the biggest killers of 2020, which resulted in 5,817,385 confirmed cases and 362,705 deaths through May 30, 2020 (Stokes et al., 2020). Pulmonary diseases impact tissue structure (Eskandari et al., 2013; Burgstaller et al., 2017; Copot et al., 2017), which in turn modifies respiratory mechanics (Faffe and Zin, 2009; Eskandari et al., 2018) in terms of conducting lung volumes (Broussard et al., 2006; Brown et al., 2006; Limjunyawong et al., 2015a,b; Robichaud et al., 2017), mechanical and viscoelastic properties (Bates et al., 2007; Suki and Bates, 2011) and air flow distribution in the lung (Rouby et al., 2003; Grasso et al., 2008). Understanding each of these changes and how they differ in healthy and diseased patients is necessary to advance diagnosis and treatment strategies in lung disease (Sattari and Eskandari, 2020).

Changes in conducting lung volumes have been used as an indicator for detecting functional changes due to disease emergence, such as fibrosis and asthma (Broussard et al., 2006; Brown et al., 2006; Limjunyawong et al., 2015a,b; Robichaud et al., 2017). Increasingly, whole lung organ displacement-loading tests, measured as volumes and pressures resulting from inflation and deflation, are used to assess lung behavior. Pressure-volume curves (PV) have been reported for various mammalian species for many years (Neergaard, 1929; Hildebrandt, 1969; Limjunyawong et al., 2015b). However, previous studies were performed using manual pressure-controlled devices with either discrete step inflation-deflation methods or pumps capable of continuous inflation-deflation (Limjunyawong et al., 2015b). As a result, these curves are not standardized, since the important factors affecting the curve shapes, including initial and maximum volume, initial and maximum pressure, and the inflation-deflation rate, vary widely across different studies and make interpretation impossible (Limjunyawong et al., 2015b). There are recent efforts to report more comparable curves using more accurate manual or commercial automatic devices (Broussard et al., 2006; Lu et al., 2006; Vanoirbeek et al., 2010; Limjunyawong et al., 2015b; Robichaud et al., 2017). However, the devices used in these studies are pressure-controlled, and measure volume change from either a known initial volume measured before the experiment or zero volume after lung degassing (Horie and Hildebrandt, 1971; Smith and Mitzner, 1980; Limjunyawong et al., 2015b). These steps demand increased preparatory efforts for testing and in the case of lung degassing, would cause the alveoli to entirely collapse, which would require much higher pressures to reopen (McLaughlin et al., 2012). Hence, providing comparable VP (volume-controlled, pressure-measured) curves starting at a known consistent pressure without any need for initial lung measurement or degassing would improve accuracy and reduce experimental challenges. Furthermore, by switching from a pressure-controlled mode to a volume-controlled device, new and currently unexplored material features, such as viscoelasticity, can be evaluated.

The lung has been deemed a viscoelastic material given it exhibits static hysteresis and stress relaxation (Bayliss and Robertson, 1939; Mount, 1955; Butler, 1957). The remodeling of pulmonary tissues and change in viscoelastic properties are expected in pulmonary disease progression but the underlying mechanisms are still not understood (Suki et al., 2005; Suki and Bates, 2011; Eskandari and Kuhl, 2015; Eskandari et al., 2015, 2016). Despite its importance, dated studies attempted to consider viscoelastic properties but were greatly limited to manual techniques and empirical descriptors (Mount, 1955; Butler, 1957; Hughes et al., 1959; Marshall and Widdicombe, 1961; Hildebrandt, 1969; Lorino et al., 1982); these experiments appear to have been done using a syringe pump, which make the measurements inaccurate and causes data loss (Robichaud et al., 2017). Therefore, a novel automated device, which controls volume and measures pressure, can offer unique measurements of viscoelastic and temporal properties, and can provide improved detection of material behavior in healthy and pathological conditions.

Additionally, various lung diseases exhibit alterations to pulmonary airflow distribution due to heterogeneous expansion caused by reduced air spaces (Grasso et al., 2008; Faffe and Zin, 2009); this decreases effective lung volume, increasing the risk of overventilation and is one of the reasons that ventilator induced lung injury (VILI) occurs (Gattinoni and Pesenti, 2005; Grasso et al., 2008; Faffe and Zin, 2009; Beitler et al., 2016). The disadvantage of modern ventilators is attributed to positive-pressure delivery to the lung, in contrast to the negative-pressure ventilation characteristic of natural breathing (Diaz and Heller, 2020). Contracting COVID-19 can induce acute respiratory distress syndrome (ARDS), where the need to ventilate exacerbates irreversible and potentially fatal pulmonary injuries in ARDS patients (Amato et al., 1998; Li and Ma, 2020). The drastic uptick in the number of ventilated patients due to COVID-19 necessitates finding improved ventilator performance to reduce pulmonary forces to physiological levels. This can be accomplished by directly analyzing and comparing lung tissue under both artificial (positive-pressure ventilation, PPV) and physiological (negative-pressure ventilation, NPV) breathing mechanics.

To address these critical needs in the pulmonary science community, here we introduce a newly constructed volume-pressure ventilation system controlled via interfacing software for experimental tissue testing in the laboratory that surpasses the capabilities of traditional ventilators by enabling viscoelastic measures and direct comparison of positive- to negative-pressure ventilation mechanics. This device provides a standardized VP curve through the application of a known pressure datum state, eliminating the need for lung degassing. Continuous volume control and pressure measurements enables novel observations regarding tissue relaxation at the organ-scale; the design also facilitates topological strain measurements of the whole lung for the first time using digital image correlation. Validation is conducted using various materials, including *ex vivo* mice lungs and latex elastic balloons, and device performance is compared to existing literature to evaluate accuracy. Other viscoelastic features including hysteresis, preconditioning sensitivity, as well as rate and strain dependency are assessed. Lastly, the device’s dual-piston scheme is examined using the same water bladder subjected to the mechanics of forced air influx versus vacuum effect, to demonstrate the artificial positive-pressures of modern ventilators versus physiological negative breathing pressures.

## Methodology

### Conceptual Design and Machine Construction

The design considerations of this machine included volume-control with recorded pressure in order to enable novel whole lung viscoelastic measurements, rate-dependency insights, and direct positive- and negative-pressure ventilation comparisons, all while accounting for air compressibility. The control objective was to equilibrate the pressure inside the lung or the pressure inside the tank in real-time depending on positive- versus negative-pressure mechanics settings, inspired by the physiology of breathing. The *ex vivo* specimens would be free to expand by suspending the specimen on a liquid layer to minimize friction during deformation. Additional design considerations included a transparent tank to allow digital image correlation for topological strain measurements (Mariano et al., under review^1^).

**FIGURE 1:**
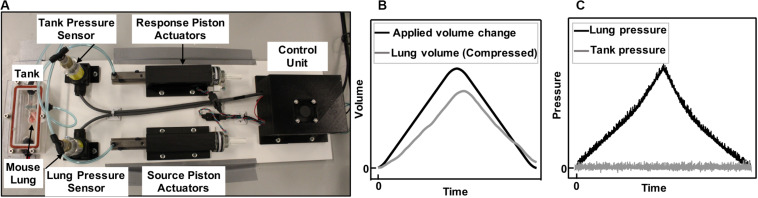
Experimental device capable of inflating and deflating the whole lung **(A)**. Components included airtight, transparent tank, control unit, and tubing connecting the lung placed inside the tank to the source piston and actuator, as well as the outlet of the tank to the response piston and actuator. Positive-pressure ventilation representative plots of applied and recorded volume change to a mouse lung **(B)** and the resulting rise in lung pressure and controlled zero change tank pressure **(C)** is shown. Air was pushed inside the lungs (**B**, black line) and the lung pressure rises (**C**, black line). As the lungs expanded in the tank, they caused a rise in the tank pressure due to decreased available tank space. The tank response piston, which recorded the pressure in real time, moved to make more volume available in the tank and maintain atmospheric pressure (**C**, gray line). The change in volume of the lung was therefore recorded through the response piston actuator’s motion, enabling the recording of the lung’s compressed air volume in real time (**B**, gray line).

In collaboration with CellScale Biomaterials Testing, Inc. (Waterloo, Canada), the device was constructed as shown in Figure 1A with a small tank (100 mm × 40 mm × 27 mm) for mouse and rat lung specimens; an analogous system with large tank dimensions (474.6 mm × 374.6 mm × 274.6 mm–not shown) was also constructed for porcine and human lung testing. The system applied and controlled volume air displacement using the source and response pistons coupled to two separate actuators. These piston actuators (23Y204S-LW8S, Anaheim Automation Inc., Anaheim, CA, United States) were connected to the lung and the tank using two outlets on the airtight, transparent tank. Movement of piston actuators would push air in or extract air out of the closed system, which changed the lung volume and led to a pressure imbalance. Two pressure sensors (SS312 series sensor, Sendo Sensor, Huangshan, China) located at the lung entrance and inside the tank would record pressure changes in real time, moving the response piston to control the zero change (net atmospheric) pressure control objective.

We controlled the mechanical device via C++ custom software, which allowed the user to define the desired testing protocol using an applied volume change, preconditioning and testing cycles, preload pressure, proportional-integral-derivative (PID) setting variables, inflation/deflation duration, hold, recovery and rest. The maximum inflation capacity for the systems are 3 ml and 3 L for the mouse and pig systems respectively; similarly, the fastest inflation/deflation rates are limited to 3 ml/s and 1 L/s.

Preloading design was a necessary step for comparable datum states across all samples regardless of trapped air inside the lung. The specimen was inflated to the preload pressure before recording volume-pressure responses but after the resetting step was achieved. System resetting was performed to set the piston actuators in a known reference starting point and vent the system to ensure atmospheric pressure by opening both lung and response outlets. Then, the lung outlet was connected to the source piston actuator and the specimen was preloaded to a defined pressure while the response outlet was still open and caused no changes to the response piston.

Once the preload was attained, the response outlet was closed to seal the system and enable active control of the tank pressure. The testing protocol was initiated by movement of the source piston, which inflated the specimen to a predefined maximum volume and at a predefined inflation rate according to the testing protocols. Simultaneously, the response piston actuator countered the pressure imbalance using a proportional-integral-derivative (PID) controller: as the lung was inflated (Figure 1B, black line) the lung pressure rose (Figure 1C, black line). The available volume inside the tank shrunk due to the lung expansion, and this caused a rise in tank pressure; since the tank pressure was being controlled to atmosphere (Figure 1C, gray), the response piston was cued to make more tank volume available; this change in tank volume was the true measure of lung volume, which was expected and found to be lower than the applied volume (Figure 1B, gray) because of the compressibility of air. This method was a direct measurement of air compressibility, in real time, counter to previous studies that applied numerical multipliers or suggested a constant post-calculation correction to account for air compressibility manually (Limjunyawong et al., 2015b; Robichaud et al., 2017).

A PID controller is a feedback control loop which continuously calculates an “error value” defined as the difference between the atmospheric pressure (desired target) and the pressure reading received from the tank pressure sensor (measured process variable). Then the calculated error was used to correct the speed and direction in which the response actuator and piston need to move to add or remove volume to maintain atmospheric pressure (Figure 1C). The correction is calculated based on the PID values determined by the user. Once values were determined by multiple preliminary trial and error experiments to minimize observed noise in the zero-pressure response, the same values were used for all subsequent tests. The best control performance for all our testing materials was found at *P* = 70, *I* = 0, and *D* = 0.

In addition to the ventilator capabilities, which applied positive-pressure to the lung specimen, this uniquely designed system replicated natural breathing by inverting the role of the source and response pistons, and induced negative-pressure ventilation. Similar to historical “iron lungs,” this negative-pressure system employed one piston to act as a diaphragm, which induced a pressure drop in the tank because of an applied increased tank volume. In this configuration, the lung pressure acted as the control to the pressure imbalance and was equilibrated to atmospheric pressure by allowing air into the lungs from the newly renamed response piston.

### Sample Preparation and Experimental Protocol

**FIGURE 2:**
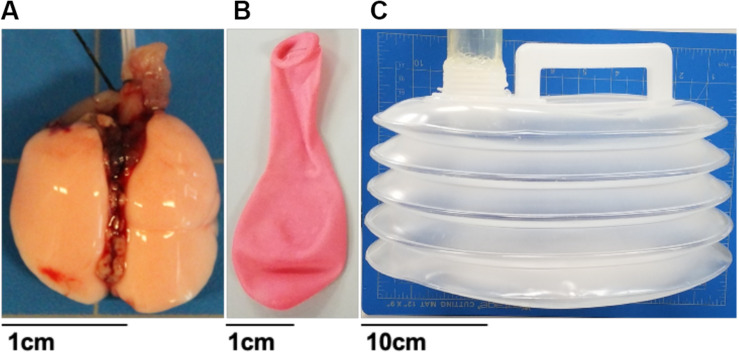
Various tested material specimens ranging from viscoelastic mice lungs **(A)**, elastic materials such as a balloon **(B)** and collapsible accordion water bladder **(C)**. Balloon and mice lungs were compared to investigate the system’s volume-, rate-, and viscoelastic-dependent capabilities. The bladder was used to assess the systems’ ability to replicate positive-pressure ventilation or physiologically representative negative-pressure breathing.

To demonstrate and validate the capability of this ventilator system, several materials were tested including 8-week old C57BL/6 mice lungs (purchased through Jackson Laboratories, approved through the University of California at Riverside Institutional Animal Care and Use Committee AUP#20170030, originally dated 8/15/2017). Prior to experimentation, animals were housed in a barrier facility with *ad libitum* access to food and water and under a 12-h light/dark cycle. Two representative mice lungs (0.16 g), a small biodegradable rubber latex balloon and a 5L collapsible accordion water bladder (Figure 2) were examined. Specimens were subjected to protocols designed for comparisons to previous mice studies, in addition to obtaining viscoelastic behaviors and direct positive- and negative-ventilation mechanics.

After anesthetization and sacrifice, a cannula (20 gauge) was inserted into the mouse trachea and inflated using a syringe (1 ml) to avoid collapse during lung extraction. The cannula was secured to the trachea with medical thread. The lung tissue with attached syringe was stored in 1× PBS and testing was performed within 4 h after sacrificing to minimize biological changes.

**FIGURE 3:**
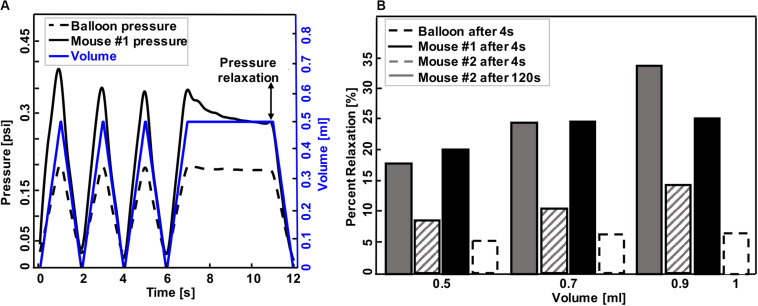
A representative graph of volume-time protocol for mouse #1 and balloon and recorded pressure-time response **(A)**. Comparable percent relaxation for mice lungs and balloon at different maximum volumes and holding durations **(B)**. Rate of inflation-deflation for balloon and mouse #1 was 0.5, 0.7, and 1 ml/s for inflation volumes of 0.5, 0.7, and 1 ml, while the rate of inflation for mouse #2 was a constant value of 0.1 ml/s for all inflation volumes. Balloon and mouse #1 were held for 4 s, while the holding time for mouse #2 was 120 s. Minimum pressure values were determined after 4 and 120 s. As expected, the mouse lung exhibits preconditioning dependency and viscoelastic pressure-relaxation when the volume is held constant over time.

**FIGURE 4:**
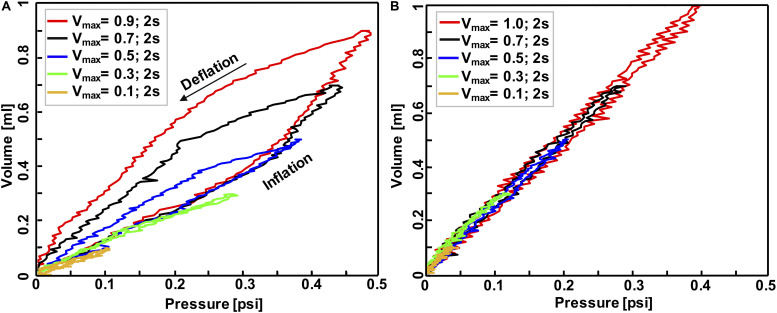
Volume-pressure curve of the third inflation-deflation cycle in mouse #1 **(A)** and balloon **(B)** where both maximum inflation-deflation volume and rate was variant: applied volume ranged from 0.1 to 1 ml while the whole inflation-deflation cycles has a duration of 2 s across all experiments. These tests confirmed the system was capable of capturing the expected elastic behavior of the balloon versus inelastic behavior of biological tissues.

Experimental testing protocols are listed in Table 1. Different sample materials were examined to validate device functionality and explore novel data collection capabilities. Dependent variables, including pressure, hysteresis, compliance, and pressure relaxation, were recorded in response to changes in volume and inflation-deflation rates. Specimens were suspended in the tank filled with 1× PBS and preloaded by inflating until the pressure reached 0.05 psi. The balloon and mouse #1 were inflated in 1 s to volumes of 0.1, 0.3, 0.5, 0.7, 0.9, and 1 ml then deflated at the same rate. In order to assess accuracy and reproducibility of the device, detect any possible air leaks, and establish preconditioning protocols necessary for biological tissues, three consecutive inflation-deflation cycles were performed and deemed sufficient for obtaining a reproducible response (Figure 3A; Robichaud et al., 2017). The fourth inflation was used to measure the viscoelastic pressure-relaxation response held at a specified inflation volume for 4 s (Figure 3B). The volume-pressure response from the third inflation-deflation cycle was analyzed (Figure 4).

**TABLE 1:** Experimental protocols used on various testing materials in this study.

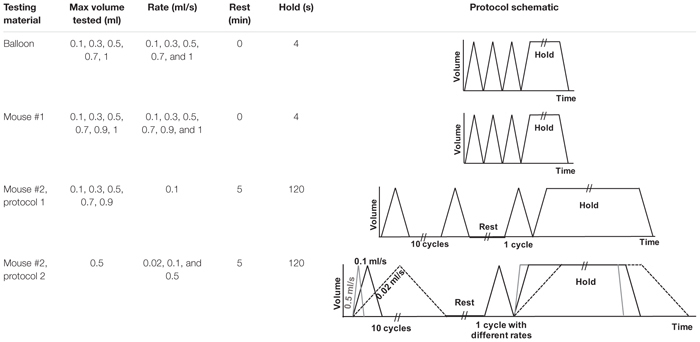

**FIGURE 5:**
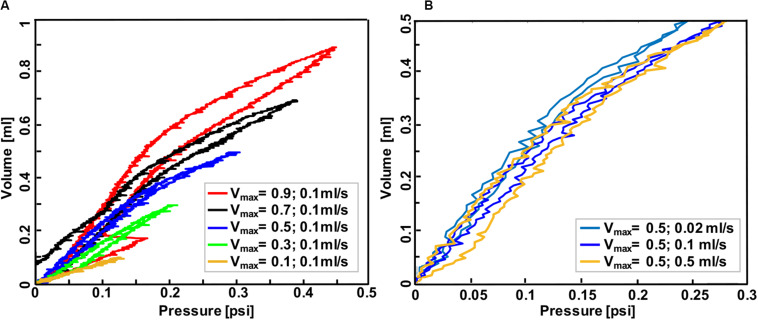
Volume-pressure curve of the third inflation-deflation cycle in mouse #2 lung where the maximum volume ranged from 0.1 to 0.9 ml with a constant rate of 0.1 ml/s **(A)** and where the inflation volume was fixed at 0.5 ml and the inflation-deflation rate varied **(B)**. Less compliant behavior was found when the lung was inflated to smaller volumes at a fixed rate, and also when it was inflated faster to a specified volume.

Mouse #2 was tested in two different settings to parse out the volume versus rate dependency of the specimen: in the first protocol, the effect of volume was investigated by maintaining a fixed inflation rate of 0.1 ml/s and varying the maximum applied volume to 0.1, 0.3, 0.5, 0.7, and 0.9 ml (Figure 5A). The second protocol maintained a maximum applied volume of 0.5 ml and explored the effect of inflation rates at 0.02, 0.1, and 0.5 ml/s (Figure 5B). In order to confirm preconditioning was achieved after the third cycle, this mouse lung underwent 10 inflation-deflation cycles but data obtained from the 3rd cycle was analyzed for comparative states. After 300 s rest, one inflation-deflation cycle and one inflation with subsequent 120 s hold was performed to measure the pressure-relaxation response at 4 and 120 s (Figure 3B; Carew et al., 2000; Noble et al., 2005).

Hysteresis was defined as the area between the loading and unloading curves (Figures 4, 5). Percent relaxation was calculated as the ratio of pressure reduction during sample holding to peak pressure (maximum minus minimum pressure value after 4 s for balloon and mouse #1, as well as 4 and 120 s for mouse #2, Figure 3B). Material compliancy is qualitatively defined as the tangent of the volume-pressure curve, increasing with increasing slope and noting the non-linear behavior. Mechanical stiffness is defined as the inverse of compliancy. The applied volume is represented in the VP curve results.

**FIGURE 6:**
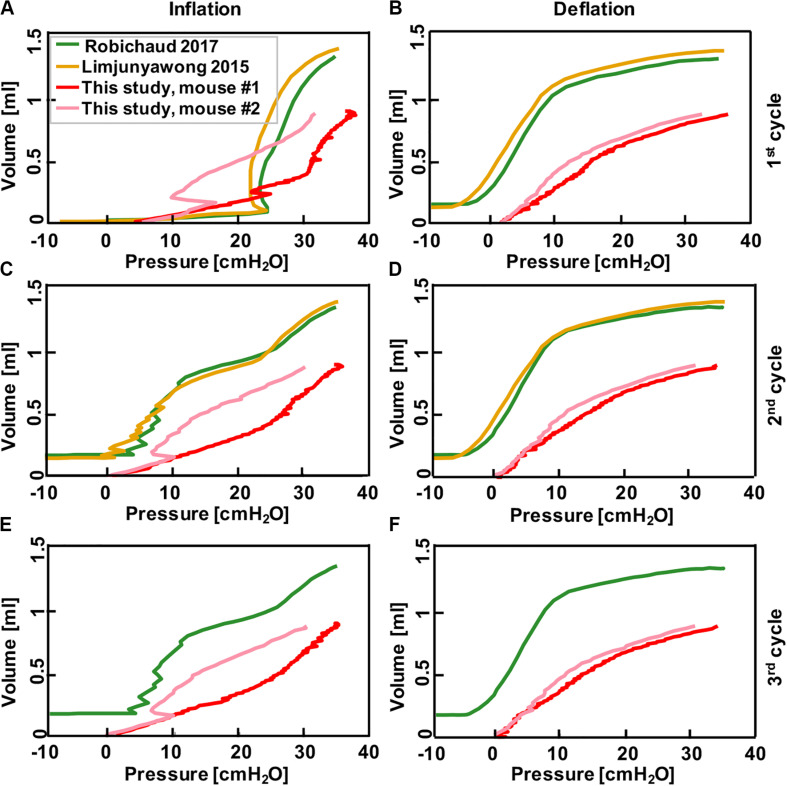
Comparison between results collected from pressure-control systems in the literature where mice lungs were inflated to 35 cmH_2_O after degassing (Limjunyawong et al., 2015b; Robichaud et al., 2017), and the results obtained from this current study where mice lung were inflated to volume of 0.9 ml using the volume-controlled device. Graphs show continuous volume-pressure curves during inflation in first, second, and third cycles **(A,C,E)** and corresponding deflations **(B,D,F)**, respectively. Rate of inflation-deflation were 0.083, 0.05, 0.9, and 0.1 ml/s for Robichaud et al. (2017) and Limjunyawong et al. (2015b), mouse #1 and mouse #2, respectively. Despite the significant differences in tissue preparation and loading techniques in these studies, results demonstrate comparable inflation-deflation trends.

In order to validate this device against existing devices in the literature (Limjunyawong et al., 2015b; Robichaud et al., 2017), the volume-pressure curves were compared over the first three inflation-deflation cycles. Devices used in both previous studies were subject to different settings given those systems controlled for pressure instead of volume: mice lungs were inflated with the rate of 0.083 ml/s (Robichaud et al., 2017) and 0.05 ml/s (Limjunyawong et al., 2015b) until they reached the pressure of 35 cmH_2_O while volume changes were recorded simultaneously. The 0.9 ml volume inflation reached similar pressures to these previous studies and the comparative results are shown in Figure 6. The system used in Limjunyawong et al. (2015b) study is a manual device and C57Bl/6 mice were used, while Robichaud et al. (2017) used a commercial automatic device “FlexiVent FX system (Scientific Respiratory Equipment SCIREQ, Montreal, QC, Canada)” and BALB/cJ mice.

**FIGURE 7:**
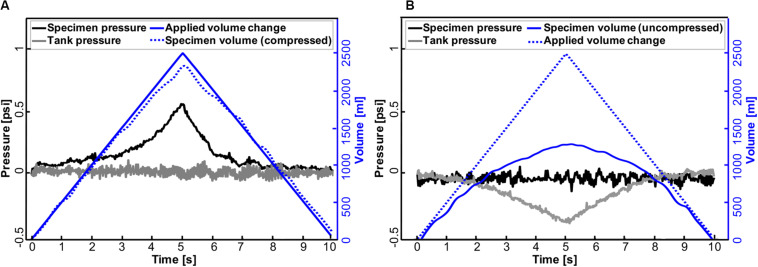
Representative pressure-time response of the accordion bladder and tank to volume changes in positive- **(A)** and negative-pressure ventilation **(B)** at a constant inflation-deflation rate of 500 ml/s. Changes in air volume applied to the bladder (PPV) and tank (NPV) are mirrored in bladder pressure and tank pressure, respectively.

Existing literature only allowed comparisons with positive-pressure included mice specimens; therefore, the PPV and NPV capability of this device was evaluated using the collapsible accordion water bladder with expected elastic behavior (Figure 2). The protocol conducted in the large tank system was as follows: after reaching a preload of 0.05 psi in PPV, the bladder was inflated to 2500 ml at a rate of 500 ml/s and deflated at the same rate. Comparatively, in NPV, the preload pressure was set to −0.05 psi and 2500 ml air was extracted from the tank at 500 ml/s. For both PPV and NPV, tests were cycled twice as the elastic nature of the bladder did not require preconditioning, and the results from one cycle are shown (Figure 7).

## Results

### Device Validation and Assessment

Representative volume-time and pressure-time curves for the balloon and mouse #1 inflated to 0.5 ml were shown in Figure 3A. The device yielded expected material behavior measurements for elastic (latex balloon) and viscoelastic materials (mouse lung): the mouse lung demonstrated preconditioning dependence, varying in peak pressure values for each inflation and deflation cycle (solid black line); in contrast, the elastic balloon’s peak pressure value was nearly identical for each cycle (dotted black line) in response to the cyclic volume change (blue). Additionally, the inert elastic balloon yielded minimal variation in the pressure over time when the inflation volume was held constant; on the other hand, the viscoelastic mouse lung’s pressure dropped from 0.33 to 0.29 psi (Figure 3A).

The expected differences between elastic and viscoelastic materials were also measurable via the pressure-relaxation response due to different maximum inflation volumes and inflation rates during loading (Figure 3B): the balloon showed negligible increase in pressure relaxation values for simultaneous increase in maximum inflation volumes and inflation rates (dotted black line); conversely, mice lungs relaxed more when larger inflation volumes were used at faster rates (mouse #1, solid black) or when solely the volume increased (mouse #2, solid and lined gray). Among mice lungs, mouse #1, which inflated faster to the same maximum volume as mouse #2, showed a higher relaxation amount compared to mouse #2 when they both held for 4 s. Holding mouse #2 volume for 120 s (solid gray) resulted in higher pressure reduction compared to shorter holding times of 4 s (lined gray).

The accuracy, repeatability, and airtight nature of the machine was confirmed by cyclically inflating and deflating several materials ranging from elastic to viscoelastic biological materials, resulting in closed-loop volume-pressure measures (Figure 4). The effects of maximum inflation volumes and inflation rates were examined and different behaviors of elastic and viscoelastic materials were anticipated: the mouse specimens demonstrated increased hysteresis (Figure 4A), whereas the balloon’s hysteresis was unaltered by the maximum applied volume as well as the inflation rate, and it showed minimal variation between loading and unloading volume-pressure curves (Figure 4B); mouse samples also exhibited more compliant behavior in response to volume and rate increases (Figure 4A), while a constant stiffness response was found for the inert elastic balloon.

Mouse #2 was used to assess the role of maximum volume and inflation rate, and to further assess the device sensitivity in recording viscoelastic features (Figure 5): increased maximum inflation volume, with a fixed inflation rate of 0.1 ml/s, resulted in more hysteresis and more compliant tissue behavior (Figure 5A); on the other hand, when the maximum volume was held constant at 0.5 ml, more compliant behavior and lower pressures were recorded when samples were inflated slowly (0.02 ml/s), compared to medium (0.1 ml/s), and fast (0.5 ml/s) inflation rates.

### Comparison With Previous Literature

After classical biomechanics concepts were used to validate this device, we compared the continuous volume-pressure curves obtained in this study to previous mice studies and found good agreement despite differences in settings, starting point and testing protocols (Figure 6). In those studies, a volume increase was observed as a result of pressure rise in inflation cycles (green and yellow; Figures 6A,C,E); similar trends have been found in this study with the difference that we observed pressure rise as a result of volume increase (pink and red; Figures 6A,C,E). Within each study, the shape of second and third inflation cycles trended similarly, while the first inflation cycle differed due to tissue conditioning. The first and subsequent deflation curves were quite similar between this study and that of previous works (Figures 6B,D,F).

### Positive- Versus Negative-Pressure Ventilation

Further analysis was done to assess the capability of this device in implementing artificial (positive-pressure; Figure 7A) and physiological (negative-pressure; Figure 7B) breathing mechanics. As can be seen in PPV, the specimen pressure rises with increased applied air volume delivered to the bladder. Peak pressure was attained at 0.54 psi at a maximum volume of 2500 ml (Figure 7A). Meanwhile, the tank pressure was equilibrated as its connected piston moved to record the volume of compressed air that the bladder actually received in real time. In contrast, in NPV, changes in air volume occurred in the tank where the same amount of 2500 ml air was extracted from the tank resulting in a tank pressure drop of 0.37 psi. At the same time, the bladder expanded (Figure 7B), and its connected piston kept the pressure in the specimen equilibrated.

## Discussion

A novel lung macromechanical device is constructed and evaluated in this study. The validated apparatus has the ability to automatically inflate and deflate the whole lung using air displacement, aiming to gain continuous, reproducible volume-pressure measurements, examine viscoelastic features quantitatively, and directly compare artificial and physiological breathing mechanics measures for the first time. The device yields similar volume-pressure curves from the literature without the need for lung degassing, mitigating potential mechanical changes due to collapsed airways caused by the removal of residual air volume (Limjunyawong et al., 2015b; Robichaud et al., 2017).

The absent sensitivity of the balloon to preconditioning, pressure relaxation, hysteresis, and applied strain levels in contrast to remarkably dependent VP responses noted in the mice lungs, confirms the adherence of this device to classical biomechanics theorems (Figures 3–5). The observed hysteresis in our experiments has been extensively noted in previous literatures in human lung (Butler, 1957; Mead et al., 1957) and experimental animal lungs such as rabbit, cat, monkey and dog (Bernstein, 1957; Mead et al., 1957; Hughes et al., 1959; Bachofen and Hildebrandt, 1971). The surface tension is claimed to be the main cause of hysteresis, since air-filled lungs exhibit this behavior, while saline-filled specimens mute this behavior (Mead et al., 1957); however, the hysteresis in saline-filled lungs still exists, confirming that hysteresis is also a tissue property and not solely due to surface forces (Hughes et al., 1959). On the other hand, hysteresis has also been related to irregular expansion of air-filled lungs; Radford (1957) reported that closed bronchioles and alveoli will open during inflation and will remain open during deflation, which is one of the reasons why hysteresis occurs.

The dependencies of hysteresis, compliance, and pressure relaxation on maximum inflation volume and inflation rate have been studied as disease indicators of viscoelastic tissues. The observed hysteresis of mice lungs in small volumes of 0.1 and 0.3 ml is minimal (Figures 4A, 5A), similar to the elastic balloon behavior at all tested volumes; however, the exhibited hysteresis increases with increasing inflation volumes, in agreement with previous studies performed in human and dog lungs (Bayliss and Robertson, 1939; Alfrey, 1948; Mead et al., 1957). Moreover, the inflation rate has also been reported to have a unidirectional but marginal effect on the hysteresis (Bayliss and Robertson, 1939; Hughes et al., 1959), which is also shown in this current study (Figure 5B). In terms of compliance, various studies demonstrate that breathing frequency does not affect normal lung behavior, but impacts diseased states: the compliance decreases with increasing breathing frequency in patients with asthma and emphysema, as well as in normal lungs with induced bronchospasm (Otis et al., 1956; Grimby et al., 1968; Seaton et al., 1972; Mitzner, 2011). While these studies were performed on patients using a plethysmograph or spirometer, an excised cat lung study similar to this experimental setup also found that compliance is rate-dependent (Hildebrandt, 1969); our preliminary tests similarly find compliance decreases with increasing inflation rate in mouse #2 (Figure 5B). Compliance and inflation volume also show a unidirectional trend in this work: as the inflation volume increases, the volume-pressure response becomes more compliant. Pressure-relaxation also increases with increasing inflation volume (Figure 3B), which is in agreement with Hughes et al. (1959).

This apparatus is further validated against previous PV mice studies conducted on automatic pressure-controlled devices (Figure 6). The general trend and shape of the volume-pressure curves and the pressure range are similar despite differences in tissue preparation techniques. Additionally, while the first inflation cycle is not comparable, the second and third cycles trend better; the difference in the first cycle has been attributed to the opening of previously closed pulmonary units or alveoli due to degassing methods used in these studies (Limjunyawong et al., 2015b; Robichaud et al., 2017). Inflating the lungs from a collapsed state requires more opening of closed airways, and lung maintains this greater capacity during the first deflation which makes the next inflation cycle start at a higher capacity state (Bernstein, 1957; Butler, 1957; Mead et al., 1957; Hughes et al., 1959). Unlike inflation, deflation is more uniform and all three cycles trended well across the studies.

Despite similarities, the variations in these results from previous studies may stem from the wide variations in testing conditions (Salmon et al., 1981; Soutiere and Mitzner, 2004; Mitzner, 2011). Firstly, excised mice lungs are examined in this study, while Robichaud et al. and Limjunyawong et al. examine mice lungs with intact chest walls immediately after absorbing 100% oxygen (Limjunyawong et al., 2015b; Robichaud et al., 2017). This may explain the double slope feature of prior works, which is less pronounced in the VP curves seen here. Secondly, the initial volume and pressure states are different between this and previous work, offsetting the curves; the degassed lungs in prior studies start from a negative pressure, while we apply a constant pressure of 0.05 psi to establish a consistent starting point and consider the starting lung volume to be at this point. Additionally, the inflation-deflation rate used here is faster (1 ml/s) compared to those studies (0.083 and 0.05 ml/s), designed to better replicate physiological breathing states (Limjunyawong et al., 2015b; Robichaud et al., 2017).

Accounting for gas compressibility is a critical step in the device setup, since gas volume decreases with an increase in pressure, making the actual applied volume delivered to the lung/tank less than the movement of the piston. This important feature is accounted in this device through real time measurement of the response piston movement. The gas correction has been performed in previous studies with different methods, such as calculating a constant coefficient manually (Limjunyawong et al., 2015b); this calculated pre-experiment coefficient only works for a specific applied air volume and should be recalculated upon any volume change. Moreover, this coefficient can only be used when the lung is degassed, or the starting volume is known. In contrast, the applied method of gas compressibility correction done here does not require any preplanning preparation and is accounted for automatically during experimentation.

The other novel feature of this device is the ability to perform positive- and negative- ventilation by simply inverting the software control of the source and response pistons. For the purposes of illustrating the capabilities of this machine for this study, we have utilized the same applied volume displacement; other similar metrics may be explored in the future, including recording the response for a matching change in lung volume (compressed air) or absolute pressure magnitude in NPV and PPV. Figure 7 demonstrates that for the same applied volume of air, the change in absolute pressure does not drastically differ as air is pushed inside the specimen (PPV) or the air pulls open the specimen (NPV). However, the change in the resulting compressed air volume of the specimen differs by more than 1L between PPV and NPV, which will have implications on local tissue strains. Such insights are unique to the PPV/NPV comparative respiratory mechanics enabled by this device.

There are studies comparing the effects of these two modes of ventilation, but to the best of our knowledge, there is no similar device and equivalent method to date. Grasso et al. compared the lung behavior in two different positive and negative ventilation devices (Grasso et al., 2008). Although they used the common method of positive ventilation by delivering air to the lung directly, their negative ventilation differed from this study’s *ex vivo* lung response, as subjects were placed in a whole body chamber. They report better oxygenation and less lung injury because of better distribution of air flow as indicated by dynamic computed tomography and histology (Grasso et al., 2008). Understanding air flow distribution is important, since distribution is complex and heterogeneous in diseased lungs and might behave significantly different in positive- and negative- ventilation (Gattinoni et al., 1986; Skaburskis et al., 1987; Easa et al., 1994; Grasso et al., 2008; Arora et al., 2017).

The design of this system further integrates fast, high resolution cameras to assess lung heterogeneity via three-dimensional non-contact surface strain topology obtained from digital image correlation, allowing pulmonary deformation to also be used as a metric between PPV and NPV. The direct comparisons between artificial and physiological breathing mechanics can help to emulate natural breathing forces by adjusting ventilator protocols in patients suffering from respiratory diseases; this is a current study underway in our lab to prevent ventilator-related pulmonary injury and death, directed at the growing use of ventilators in the COVID-19 pandemic.

### Limitations

A secondary effect of collecting measurements continuously is noisy data; experiments must be prefaced with PID exploration to ensure reduction of erroneous sampling. However, the noise observed in the plastic bladder specimen (Figure 7) is resolved when testing larger porcine lungs and by removing excess volume by adding incompressible fluid to the tank, thereby decreasing the control volume necessary for equilibrating.

Since the specimens used in this study are not degassed, the full range VP curves are not reported. The tradeoff being that this method of preloading can be applied as many times needed, which allows multiple tests on one specimen to examine different factors; while Robichaud et al. reported full range PV curves, the lungs could be degassed only once (Robichaud et al., 2017), limiting experimental procedures to a single experiment on each lung.

The other limitation is the interference of air flow resistance as one of the factors affecting the viscosity force and pressure measurements. In order to alleviate this issue, a quasi-static ventilation rate is used in these experiments to minimize the flow resistive pressure. The frequency is set at or less than 30 cycles/min to abide by the thresholds reported in studies of Bayliss and Robertson (1939) and Mount (1955), who showed that in frequencies lower than 100 cycles/min, the hysteresis is reliably due to the deformation of lung structure and not air flow.

The limited number of samples in this study precludes any statistically significant conclusions regarding the mice specimens or evaluations beyond qualitative insights, and such experimental results are beyond the intended scope of this manuscript. The preliminary results shown here are to illustrate and confirm the capabilities of this newly designed device, and to provide a foundation for future procedures to utilize these methods and improve the latitude of existing pulmonary experiments.

## Conclusion

This work introduces a novel volume-controlled device that can be used in future studies to understand the changes in mechanics, viscoelasticity, and airflow distribution due to pulmonary disease emergence. These investigations ultimately enable future scientific considerations augmented by viscoelastic measures and adjustments to commercial ventilator settings based on direct assessment and comparisons of positive- and negative-pressure mechanics.

## Data Availability Statement

The raw data supporting the conclusions of this article will be made available by the authors, without undue reservation.

## Ethics Statement

The animal study was reviewed and approved by Institutional Animal Care and Use Committee approval AUP#20170030, originally dated 8/15/2017.

## Author Contributions

ME conceptualized and designed the system. SS and CM designed the experiments. SS, CM, and SV performed the experiments. JV and TN provided the resources. JP, CH, and ET manufactured the system. SS and ME analyzed the data, interpreted the results, drafted the figures, and wrote the manuscript. All authors contributed to the article and approved the submitted version.

## Conflict of Interest

The authors declare that the research was conducted in the absence of any commercial or financial relationships that could be construed as a potential conflict of interest.

## Footnotes

1 Mariano, C. A., Sattari, S., Ganjeh, M. M., Tartibi, M., Lo, D., and Eskandari, M. (2020). Novel Mechanical Strain Characterization of Ventilated Ex Vivo Porcine and Murine Lung using Digital Image Correlation. Under Review.
